# Online information acquisition affects food risk prevention behaviours: the roles of topic concern, information credibility and risk perception

**DOI:** 10.1186/s12889-023-16814-1

**Published:** 2023-10-02

**Authors:** Zhenwu You, Weizhen Zhan, Fan Zhang

**Affiliations:** 1 School of Journalism and Information Communication, Huazhong University of Science and Technology, Wuhan, Hubei Province China; 2 School of Journalism and Communication, Xi’an International Studies University, Xi’an, Shanxi Province China

**Keywords:** Preventive behaviours, Online information acquisition, Topic focus, Propensity score matching, Risk perception, Information credibility

## Abstract

**Background:**

The COVID-19 pandemic has not only brought great challenges to the global health system but also bred numerous rumours about food safety. Food safety issues have once again attracted public attention.

**Methods:**

The data were drawn from the fifth wave of the first Taiwan Communication Survey database. The respondents were selected via multistage stratified random sampling. The sampling units were townships/districts, villages/neighbourhoods and households. The sample consisted of 2098 respondents. This study first used propensity value matching to analyse the direct impact of online food safety information acquisition on preventive behaviours and examined the heterogeneous impact caused by the difference in the degree of topic attention through value matching. Hayes’ PROCESS macro model 6 was applied to confirm the mediating effect and the serial mediating effect.

**Results:**

The research results show that an increase in the frequency of the acquisition of online food safety information significantly increases individuals’ food risk prevention behaviour. However, only users with high concern about the issue are affected. The food risk prevention behaviour of users with low concern about this issue is not affected by the acquisition of online food safety information. Further analysis shows that risk perception and information credibility both play mediating roles in the impact of online food safety information acquisition on food risk prevention behaviour. Moreover, the transmission and united effects of information credibility and risk perception play a distal mediating role.

**Conclusions:**

Food risk prevention behaviours are an important topic for personal health as well as government management. Our study’s findings can provide empirical evidence for risk managers and decision-makers to reevaluate the role of the internet in food risk management.

## Introduction

In 1986, the German sociologist Ulrich Beck first identified the “risk society”, which describes people’s social insecurity and anxiety in industrial civilization. As an old saying in China goes, “Food is the most essential thing for common people, and food safety is the priority”. Food safety affects the national economy and people’s livelihood. In “China’s Comprehensive Well-off Index”, food safety was at the top for five consecutive years (2012–2017) among the ten issues of greatest public concern, even higher than topics such as housing prices, medical reform [1], and inflation. In 2018–2020, the topic of food safety remained in the top five [2]. In 2020, COVID-19 brought enormous challenges to the global health system and fuelled countless rumours about food safety [3]. Food safety issues once again attracted great public attention and returned to the top ten issues of greatest public concern [2]. In traditional agricultural society, people were mostly concerned with food and clothing. With the development of new agricultural technology and biotechnology, people’s food supply was greatly enriched. However, in the process of social development, risks also followed. Risks can be found in the processes of food production, packaging, preservation, and transportation, such as food packaging bags and food additives. In current society in which food safety receives unprecedented public attention, individuals should be encouraged to take practical actions to prevent or reduce the health hazards that may be caused by food safety issues.

If individual preventive behaviour is an important measure to address food risks, what factors influence individuals to take relevant actions? Previous health theory models, such as protection motivation theory, subjective expected utility theory, and the health belief model, have focused on perceived threat or fear assessment. These theories hold that the possibility of being affected by a risk or the severity of the impact caused by the risk are important psychological motivations for individuals to adopt health protective behaviours. However, these theoretical models generally overlook the role of communication media in risk events and present only fragments of individuals’ responses to risk events. When food risks occur, food risk information often cannot reach the public directly. Instead, intermediaries such as the media are needed for the public to obtain the latest news on the event and understand its progress to understand how to deal with food risks. Therefore, the media play a crucial role in the process of risk information diffusion and the formation of public risk perception [4, 5].

In the 1990s, the internet became widely available. Over the following 10 years, smartphones, social media, and streaming media extended and amplified the presence and aggregate functionality of the internet so that it reached the astounding level it has now [6]. According to authoritative survey data, the internet has become the main channel for the Chinese public to obtain information, with 783 million Chinese people mainly consuming news on internet platforms [7], accounting for more than half of the total population in China. More importantly, the changes triggered by internet technology in the field of information production and information dissemination are rapidly reshaping public opinion in today’s society. Online public opinion has become the main information platform that affects the public’s cognition and attitude towards social phenomena [8]. Thus, it is of great practical and theoretical significance to explore the relationship between the acquisition of online food safety information and food risk preventive behaviour. In previous media effect studies, the selection bias of research samples was a problem that was long ignored. Similarly, little is known about the heterogeneous impact of different degrees of attention to this topic.

To compensate for the shortcomings of previous studies, this study attempts to predict food risk preventive behaviour with a more complete theoretical framework and to examine how individuals cope with food risks. Based on the protective action decision model, this study systematically explores the structural relationships among online food safety information acquisition, risk perception, information credibility, and food risk preventive behaviour. This study utilizes data from the fifth wave of the first Taiwan Communication Survey database of 2098 Taiwanese people to explore the relationship between online food safety information acquisition and food risk preventive behaviour and construct a relatively complete research model with information credibility and risk perception as mediating variables.

Overall, this study has three contributions. First, this study focuses on the predictive effect of online food safety information acquisition on food risk preventive behaviour and uses a propensity score matching method to eliminate systematic bias between the control group and the experimental group in the matched confounding variables [9]. This addresses the issue of selectivity, solving the bias in the research results and the “net” effect of independent variables on dependent variables, which makes the research results more credible. Second, compared with the previous literature’s neglect of the impact of topic concerns on the behaviour of information acquirers, this study makes a more detailed distinction between individuals’ online food safety information concerns to provide empirical evidence of the impact of online food safety information acquisition on food risk preventive behaviour. Third, this study regards information credibility and risk perception as important intermediary variables for transforming online food safety information acquisition into actual risk preventive behaviours. More importantly, this study innovatively proposes that information credibility and risk perception should be regarded as a continuous reaction process because the stimulation effect of online food safety information exposure is not only realized through information credibility or risk perception; it is likely to affect participation behaviour through the transmission and joint effect of the two. Ultimately, the theoretical chain of “information acquisition - information credibility - risk perception - preventive behaviour” is formed, which enriches and expands the protective action decision model. This chain logic relationship has decision-making reference value for emergency management departments to formulate accurate food risk communication strategies and to prevent, control, and eliminate food risks.

## Literature review and research hypothesis

### Protective action decision model

Various theoretical models of psychological motivation and behaviour decision-making provide useful explanations for how risk communication affects disaster response and individual behaviour. For example, psychodynamic theory provides a psychological research perspective and general direction for individual social behaviour decision-making and behaviour formation. The protective motivation theory (PMT) proposed by Rogers et al. also illustrates the role of psychological regulation on behavioural performance. However, their research is more focused on risk research situations. They consider information about individual characteristics and external environmental information as triggers for protective motivation and risk assessment and coping assessment as intermediate action mechanisms for protective motivation mechanisms. Finally, individuals produce self-protective thoughts and behaviours [10]. Based on previous research models, Lindell and Perry [11] proposed the Protective Action Decision Model (PADM) and integrated the information processing process in 2012 to modify and improve the original model. Hence, the PADM is recognized as a multistage behavioural decision-making theoretical framework.

This theory states that individuals with different characteristics (such as skill use, cognitive ability, and economic resource ability) receive risk information about environmental and social factors from various information channels, which promote public attention and understanding of risk information. This triggers perceptions of risk, stakeholders and protective behaviour. On this basis, behavioural decisions are made, and corresponding protective actions are taken to reduce risks [12]. Risk perception is the perception of the possible occurrence of risk and its consequences. Protective behaviour perception is the perception of the effectiveness and cost of behaviour when taking protective behaviour. Stakeholder perception is the perception of the professionalism, reliability, and responsibility of the information source [13]. Therefore, risk perception and information credibility are important psychological motivations for individuals to receive risk information and influence their risk response behaviour.

The PADM provides the basis for explaining individuals’ behavioural decision-making processes in risky situations. It is widely used in research on protective behaviour in natural risk situations such as earthquakes [14], hurricanes [15], volcanoes [16], and floods [17]. Individuals’ preventive behaviour decision-making processes for food safety risk event situations are highly similar to that in disaster situations. When individuals with subjectivity and relative rationality are exposed to food safety risk information, they actively utilize a variety of information channels and knowledge to assess the credibility of the risk information and form risk awareness and perception to adjust their own food risk prevention behaviour. Therefore, the prevention behaviour model provides a powerful reference for the theoretical framework of this study. However, in today’s new media environment with diversified media channels and abundant information content, the public’s trust in information source channels has changed. The protective behaviour decision-making model fails to fully consider the impact of information credibility on risk perception when individuals obtain information. What is the role of information credibility in public information acquisition, risk perception and preventive behaviour? Does it affect the causal model of information acquisition, risk perception, and preventive behaviour in crisis and risk communication?

Previous studies have shown that public risk perception is a process of collecting, selecting, understanding and responding to crisis information [18]. Reliable information sources help the public form a correct perception of risk. Public risk perception is an important factor that affects public decision-making for protective behaviour [12]. Information credibility and risk perception not only play an intermediary role between information acquisition and preventive behaviour but also show a chain logic relationship. Therefore, this study attempts to embed the variable of information credibility into the causal model of “information acquisition - risk perception - preventive behaviour” to form the new theoretical chain of “information acquisition - information credibility - risk perception - preventive behaviour”. Whether the PADM can address food risk scenarios also needs to be further verified. In addition, considering that each person’s attention to food safety issues is different, there may be a heterogeneous impact on the relationship between online food safety information acquisition and food risk preventive behaviour. Therefore, this study bridges the issue of concern and the protective behaviour decision-making model to comprehensively explore the relationship between online food safety information acquisition and food risk preventive behaviour, which further expands and improves upon the PADM.

The expanded PADM helps to select appropriate social cues and resources to enhance risk perception and make protective action decisions for different individuals. Emergency management departments provide good theoretical guidance on how to choose effective information dissemination methods to enhance individual food safety knowledge and preventive awareness for different populations. Once individuals obtain relevant food safety knowledge, they can take reasonable actions to enhance their own safety when food risks occur. This awareness can also improve the risk response effectiveness of emergency management departments, which is carried out synchronously. Therefore, the expanded PADM can better guide emergency management departments in developing precise food risk communication strategies for prevention and control, thereby resolving food risks.

### Online food safety information acquisition and food risk prevention behaviour

Human behaviour and thoughts are affected by the quantity and quality of available information. Channels of risk information communication play a crucial role in the generation of risk perception and behavioural intention. In fact, most people are not witnesses of risk events. When personal experience is scarce, individuals often obtain risk information through interpersonal communication and media channels. However, the social network of each individual greatly constrains the breadth and depth of interpersonal communication, which leads to limited sources and content of information acquisition. The media has broken through this limitation by spreading diverse information and knowledge to the public on a large scale. The storage, retrieval and reuse functions of the network provide more opportunities for food risk communicators. Therefore, online news media are increasingly playing the role of food safety governance actors as sources of information [19]. In particular, the rapid rise of social media has completely changed the way we communicate, share and obtain information online. Social media has become the main channel for individuals to obtain food safety information [20].

In the past 28 years, the public has witnessed the vigorous development of the internet. Since the commercialization of the internet in 1994, this new technology has expanded rapidly around the world. It transformed the monopoly of traditional media’s domination of the release and dissemination of risk information. The internet constructed an unofficial field of risk communication that gives ordinary people and other social institutions the right to speak. Furthermore, it greatly expanded the scope and speed of the dissemination of food safety issues. According to framing theory, the framework of news reports directly affects the public’s attitude. When the public is exposed to a specific information framework, their comprehension and cognition of certain phenomena will gradually tend towards the direction of the framework [21], thus changing their actual behaviour. Therefore, if an individual is exposed to relevant food safety information amid food safety events, the individual’s preventive behaviour may be triggered. Studies have shown that the public can quickly obtain food safety information through social media, strengthen their risk perception of food safety, and take preventive actions to reduce the risk of food poisoning. [22] When the media reported the occurrence of African swine fever in other countries, it caused consumers in other disease-free regions or countries to worry and reduce their purchase of pork [23]. Therefore, in risk studies of food safety issues, it is necessary to pay attention to online food safety information acquisition and explore its role and effect. Hence, we propose the following hypothesis:

H1: Online food safety information acquisition has a significant positive impact on food risk preventive behaviours.

### Differences in the degree of concern and preventive behaviours for food risk

The modernization of China’s society is described as a “compressed modernization” that accelerates the production and reproduction of risks. It also leaves no time for the management of risks [24]. Consequently, many social problems occur in the modernization process, which can be described as “risk symbiosis” in the period of social transformation. Among various social risks, food safety issues are closely related to public life, health, and well-being and are the risk events that receive the highest degree of public concern. For the general public, attention is a scarce resource. People use their energy to focus on the information they read, which in turn promotes the formation of the public’s cognitive structure for food safety issues and guides the construction of public preventive behaviour. Previous studies on individual eye tracking have found that the reading of internet information is more selective than the reading of offline news information. It is easy for individuals to focus on specific news and improve their cognition and comprehension in specific fields [25]. Thus, individuals can access and read online food safety information selectively, understand the latest progress of the event, and know how to deal with food risks. However, in cases where insufficient attention is given to food safety information, individuals may skip food safety information and avoid the opportunity to develop preventive behaviours.

Current research shows that the way individuals perceive risk and their level of concern about risk can influence their behaviour [26]. In terms of health behaviour, the degree of public attention to relevant information on social media during the COVID-19 pandemic was positively correlated with preventive behaviour [27]. Furthermore, attention to information on the internet can positively predict individual environmental behaviour [28]. Hence, the degree of attention given to issues affects the decision-making process and adjustment of individuals’ actual behaviour. In terms of food risk preventive behaviour, the impact of online food safety information acquisition on users with high attention to issues and users with low attention to issues may be significantly different. Based on the statements above, we propose the following hypothesis:

H2: The impact of online food safety information acquisition on food risk preventive behaviours is different with regard to the degree of attention given to the issue.

### Mediating effect: information credibility and risk perception

Human behaviour is the result of cognition and motivation [29]. According to the PADM, information credibility and risk perception are two important motivations for online food safety information acquisition to affect food risk preventive behaviour. Risk perception refers to an individual’s intuitive judgement and subjective feeling about the impact and severity of external objective risks under the situation of limited and uncertain information reserves. The view of the “mediatization of risk” holds that the media play an important role in the process of risk perception [30]. On the one hand, the media provide crucial information channels for the public to recognize risks [31], especially when people cannot personally experience risk events and can only understand relevant risk information through the media; the role of media information is self-evident. Mobile apps are a health intervention method, and updated information can greatly improve users’ knowledge of diseases and preventive behaviours [32, 33]. On the other hand, media can influence people’s perception of risk because individuals can collect and process relevant data [34].

As an important medium for the public to access risk information, the internet has promoted the redistribution of risk discourse. The vast amount of food risk information on the internet has strengthened the public’s “symbolic reality” experience of risk [35]. A survey of 688 South Koreans found that personal exposure to cancer information in social media was significantly positively correlated with the respondents’ cancer risk perception [36]. Other studies have found that through social media access to information related to MERS, individuals’ risk perception level was significantly improved [37, 38]. In the context of food safety, consumers’ perceptions of food safety risks determine their intentions and behaviours in purchasing these foods [39]. An empirical study of the salmon incident in Beijing’s Xinfadi in 2020 confirmed this point: the stronger their risk perception, the more consumers avoided purchasing salmon-related food [2]. Accordingly, this study proposes the following hypothesis:

H3: Risk perception mediates the relationship between online food safety information acquisition and food risk prevention behaviour.

Information credibility is crucial for effective risk communication, and the strength of information credibility directly affects the public’s willingness to engage in preventive behaviours. People who believe that information on social media is trustworthy tend to handle risk information more seriously [40]. Information credibility is affected by factors such as the subject of information sources and channels of information dissemination [41].

Studies have shown that hard news in traditional media is more credible than that in new media, and there is no significant difference in the credibility of soft news between new media and traditional media [42]. Furthermore, from the perspective of the persuasion effect, a large number of studies have highlighted the direct and positive effects of information credibility on individual behaviour [43–46]. For example, Hong et al. (2019) [47] found that in the face of earthquake threats, information credibility affects the public’s assessment of disaster severity and evacuation decisions. Yueh et al. (2022) [48] suggested that information with high credibility can reduce the uncertainty of information seekers, making them more willing to take related actions to overcome risks. Dong et al. (2018) [49] noted that high-credibility information is better able to elicit public perceptions of climate change risks in people’s personal lives and is more likely to trigger climate-related action. Accordingly, this study proposes the following hypothesis:

H4: Information credibility mediates the relationship between online food safety information acquisition and food risk prevention behaviour.

According to the PADM, the channels and frequency of obtaining information in risk events affect the information credibility and risk perception of the public, which in turn affects people’s decision-making behaviour. Is there a potential impact mechanism between the two major psychological perceptions of information credibility and risk perception? Some studies have shown that information credibility is an important predictor of risk perception [50, 51]. Studies have also shown that media messages shape people’s perception of risk and subsequently influence their mental health and behaviours [52]. Based on the above discussion, this study argues that information credibility affects people’s acquisition of information on food safety online, further affecting their subjective perception and judgement of risk and thus changing their intention to prevent behaviour. Therefore, we hypothesize the following:

H5: Information credibility and risk perception play a chain intermediary role between online food safety information acquisition and food risk prevention behaviour.

## Methodology

### Sample and data source

This study uses survey data from the fifth wave of the first Taiwan Communication Survey database. This wave of the survey took “risk and disaster communication” as the research topic and Taiwanese people over 18 years old as the interviewees. The study used a stratified three-stage PPS sampling method to sample towns and cities, villages, house numbers, and family members and obtained a total of 2098 valid samples.

### Measurement

#### Food risk preventive behaviour

Ten behaviours were measured that people adopt to protect themselves against common food risk events and serious food incidents in Taiwan. These strategies are promoted by national and local health and food safety authorities (e.g., the Ministry of Health and Welfare, Food and Drug Administration, and Office of Food Safety of the Executive Yuan), whose official websites contain relevant policies and news related to food safety.^1^ They were also promoted by consumer groups and nonprofit organizations focused on health promotion [53, 54]. Examples of items are “avoiding drinking beverages in plastic cups” and “avoiding using plastic bags and plastic containers holding cooked food or for microwave heating”. The respondents’ answer options were “0 = No, 1 = Yes”. Cronbach’s alpha for the 10 items was 0.76, indicating that the measurement was reliable. The food risk prevention behaviour variable was measured by the sum of the scores. The total score ranged from 0 to 10, *M* = 6.165, *SD* = 2.617.

#### Online food safety information acquisition

This issue was assessed with the following question: “How often do you usually obtain food safety-related information (e.g., plasticizers, cooking oil safety, contaminated food, pesticide residue) from the internet?” This self-rated measurement of information acquisition has been commonly applied in previous studies [55–58]. The respondents’ answer options were on a four-point scale (1 = never, 2 = rarely, 3 = sometimes, 4 = often). The higher the value, the higher the frequency of contact with food safety information on the internet. In the propensity score matching analysis, “never” and “rarely” were recoded as 0 and “sometimes” and “often” were recoded as 1, forming the control group and the experimental group, respectively, *M* = 2.547, *SD* = 1.156.

#### Degrees of topic attention

This issue was assessed by the following question: “Do you care about food safety?” The respondents’ answer options were on a four-point scale (1 = very indifferent, 4 = very concerned). In this study, “very indifferent” and “not very concerned” were recoded as 0, representing low issue concern, while “a little concerned” and “very concerned” were recoded as 1, representing high issue concern.

#### Risk perception

Risk perception was measured with two items: “Do you think food safety problems may affect your health?” and “Do you think food safety problems have a serious impact on your health?” The respondents’ answer options were “1 = very unlikely, 4 = very likely” and “1 = not serious, 4 = very serious”. This study summed the two items and took the average to develop a “risk perception” scale. The higher the score, the higher the risk perception of the respondents. Cronbach’s alpha of the 2 items was 0.80, *M* = 3.388, *SD* = 0.650.

#### Information credibility

Information credibility was assessed by the following question: “Do you believe the food safety information provided by the internet?” The respondents’ answer options were measured on a four-point scale from “mostly do not believe” (coded 1) to “mostly believe” (coded 4). The higher the value, the more the respondents believed the information about food safety obtained online, *M* = 3.127, *SD* = 1.045.

#### Control variables

In this study, gender, age, and education level were included in the model as control variables. Males accounted for 44.6% of the sample (0 = female, 1 = male). The gender proportion was relatively balanced. The education level was divided into seven categories, with 45.8% of respondents having college degrees or above, indicating that most respondents had a higher education level. Previous literature notes that whether the public has experienced food safety problems also affects their perception of food risks [59]. Therefore, the respondents’ experience was also included in the model as a control variable. The question “Have you or your family ever been affected by food safety problems?” in the questionnaire was used for measurement. The answer options were “0 = No, 1 = Yes”.

### Statistical analysis

In the study of media effects, there are some variables that confuse the relationship between independent variables and dependent variables, resulting in selective bias and making it difficult for researchers to directly explore the “net effects” between the two. Some studies have shown that individual heterogeneity is an important reason for the physical access gap [60–62]. In the quantitative analysis, we attempted to eliminate the influence of these competitive interpretation factors, but none of the models could do so. The existing confounding variables made the research results lose the causal interpretation effect. Given the impact caused by selective bias, the effective response method is propensity value matching. The operational logic of propensity value matching is closer to the requirements of classical random experiments under the counterfactual framework. In the propensity value matching operation, individuals from different units are matched according to the proximity of propensity value points. An individual may match with multiple individuals in different groups to form a processing group and a control group. The matched samples effectively control the selective bias caused by the confounding variables, and a “quasirandom” experiment is reconstructed to calculate the “net effect” of the independent variables on the dependent variables [63]. Therefore, this study used propensity value matching to address the impact of selection bias on the research results to ensure the reliability of the research conclusions.

This study first used propensity value matching to analyse the direct impact of online food safety information acquisition on preventive behaviours. To test the robustness of the impact effect, this study simultaneously used three propensity value matching methods, radius matching, nearest-neighbour matching and kernel matching, to conduct the empirical analysis. Furthermore, the heterogeneity impact caused by the difference in the degree of topic attention was examined through the grouping tendency value matching of topic attention. Stata 15.1 SE software was used to analyse the above two steps. Finally, to examine whether information credibility and risk perception are mediators of the relationship between online food safety information acquisition and food risk prevention behaviours, we conducted a mediation analysis using Hayes’ PROCESS macro [64]. PROCESS reflects the path analysis framework to estimate the ordinary least squares regression coefficients of every model pathway. To test the indirect effects, the bootstrapping technique (5000 samples) was applied to ensure more robust estimations than the Sobel approach [65]. Bootstrapping has greater power and minimizes type I errors by resampling subsets of data from the given dataset and then summarizing the final results from the statistical tests on these subsets [66–68]. All statistical analyses were performed using IBM SPSS Statistics 26.0 and the PROCESS macro Model 6 for SPSS.

## Results

### Direct effect test

This study constructed a logistic regression to estimate the predictive propensity using the recoded two-category online food safety information acquisition as the dependent variable. The regression results showed that pseudo R^2^ was 0.273 and − 2Log Likelihood was 2072.189, indicating that the overall compatibility of fit of the model was high and the selected independent variables had a strong predictive effect on the acquisition of information on food safety online.

Since the effective sample size of this survey was limited, propensity score matching with replacement was conducted, and parallel matching was allowed. Abadie et al. (2004) [69] suggested one-to-four matching to minimize the mean square error, so the nearest neighbour matching in this study was one-to-four matching. The average treatment effect on the treated (ATT) of the respondents was the core evaluation index of the propensity matching effect [8]. In this study, ATT was equal to the food risk prevention behaviour of the high acquisition of online food safety information group (experimental group) minus the food risk prevention behaviour of the low acquisition of online food safety information group (control group), which is the real effect of the acquisition of online food safety information on food risk prevention behaviour. The results of propensity score matching in Table 1 shows that the ATT before matching was 0.534 (t = 4.36, *p* < 0.001). However, because the sample before matching was affected by the confounding variable, the net effect cannot be obtained. The ATT result reflects the pseudocause effect produced by the independent variable and the confounding variable. To obtain the real effect of food safety information acquisition on the internet, this study adopted three methods of radius matching, nearest neighbour matching and kernel matching to conduct trend value matching. The ATT results of the three matching methods were 1.057 (t = 6.46, *p* < 0.001), 1.033 (t = 5.84, *p* < 0.001) and 1.065 (t = 6.51, *p* < 0.001), respectively, which shows that the three matching results were basically consistent. The propensity value matching results had strong robustness, indicating that the acquisition of information on food safety online has a significant positive impact on food risk prevention behaviour.

**Table 1:** Propensity score matching results

Matching method	experimental group	control group	ATT	S.E	t-value
**Before matching**	6.387	5.853	0.534***	0.115	4.63
**Radius matching**	6.426	5.370	1.057***	0.164	6.46
**Nearest neighbor matching**	6.426	5.393	1.033***	0.177	5.84
**Kernel matching**	6,426	5.362	1.065***	0.163	6.51

Note: * *p* < 0.05, * * *p* < 0.01, * * * *p* < 0.001; Radius matching: radius = 0.05; Nearest neighbor matching: one-to-four matching; Kernel matching: broadband = 0.06; S.E. stands for standard error

To ensure the validity of the propensity score matching results, the matching process must meet the balance assumption and common support assumption. Taking the nearest neighbour matching (1:4) result as an example, the balance test results are shown in Table 2. The standardized deviation of all covariates after matching was less than 10%, indicating that the test results did not reject the original hypothesis that there was no systematic difference between the treatment group and the control group. Therefore, nearest neighbour matching passed the balance test. Furthermore, radius matching and kernel matching also passed the balance test. Due to space limitations, this will not be repeated.

**Table 2:** Balance Test Results of Nearest Neighbor Matching

Covariates	Standarded bias	p
**Age**	0.6%	0.884
**Gender**	-0.9%	0.825
**Education**	-0.2%	0.966
**Experience in food safety issues**	3%	0.453

**Fig. 1:**
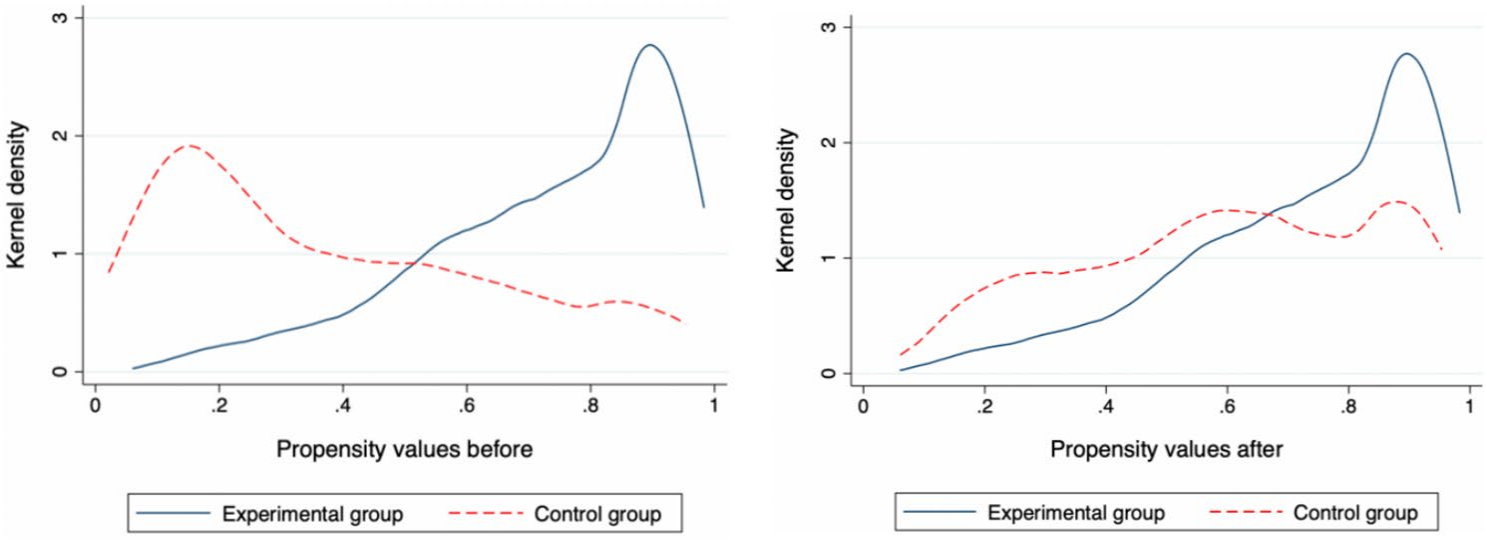
Kernel density map of propensity values before and after nearest neighbor matching

Figure 1 further illustrates the test results of the common support hypothesis of nearest neighbour matching. It can be seen from the propensity score matching nuclear density diagram in Fig. 1 that there was a very significant difference between the nuclear density curves of the experimental group and the control group. The trend of the nuclear density curve after matching was roughly the same, and the difference between the two groups of samples was significantly reduced. The existence of selective bias affected the estimation of the real effect of the acquisition of information on food safety online on food risk prevention behaviour. The situation of radius matching and core matching was similar and will not be repeated, so the three matching methods met the common support assumption. In summary, the research results of three propensity score matching are valid, and research Hypothesis H1 is supported.

### Heterogeneity analysis of topic concern

In this study, the samples with high and low topic attention were used for propensity score matching to examine the heterogeneous impact of users’ different levels of attention to online food safety information (see Table 3). The analysis of the high topic concern group showed that the ATT value before matching did not pass the significance test (t = 1.13), but after radius matching, nearest neighbour matching and kernel matching, the ATT value was 0.917 (t = 5.76), 0.866 (t = 4.81) and 0.978 (t = 5.80), respectively, and the results were significant at the 0.001 level. However, the ATT values before and after the matching of the low-topic focus group did not pass the significance test (0.04 ≤ | t | ≤ 0.7). In summary, the food risk preventive behaviour of users with low topic attention will not change with an increase in the frequency of the acquisition of information on food safety online. However, for users with high topic attention, the increase in the frequency of the acquisition of information on food safety online will significantly increase their food risk prevention behaviour. Therefore, research Hypothesis H2 is supported.

**Table 3:** The matching analysis results of the propensity value of issue attention

Matching method	Full sample		High topic attention		Low topic attention	
	ATT	t-value	ATT	t-value	ATT	t-value
**Before matching**	0.534***	4.63	0.333	1.13	-0.238	-0.70
**Radius matching**	1.056***	6.46	0.971***	5.76	-0.024	-0.04
**Nearest neighbor matching**	1.033***	5.84	0.886***	4.81	0.251	0.39
**Kernel matching**	1.064***	6.51	0.978***	5.80	-0.043	-0.07

Note: * *p* < 0.05, * * *p* < 0.01, * * * *p* < 0.001; Radius matching: radius = 0.05; Nearest neighbor matching: one-to-four matching; Kernel matching: broadband = 0.06

### Mediating effect test

We used the bootstrap method to test the mediating effect of information credibility and risk perception on the acquisition of information on food safety online and food risk prevention behaviour. The test results (Table 4) show that in the first mediation path, the acquisition of information on food safety online had a significant positive mediating effect on food risk prevention behaviour through information credibility (β = 0.007, 95% CI [0.002, 0.014], excluding 0); therefore, H3 is supported. In the second mediation path, the acquisition of information on food safety online also had a significant positive mediating effect on food risk prevention behaviour through risk perception (β = 0.043, 95% CI [0.029, 0.057], excluding 0); therefore, H4 is supported.

### Chain-mediated effect test

We used Model 6 of SPSS 26.0 as the chain mediation and adopted the bootstrap method to test the chain mediation effect of information credibility and risk perception. The test results (Table 4) showed online food safety information acquisition → information credibility → risk perception → food risk prevention behaviour (β = 0.002, 95% CI [0.001, 0.003], excluding 0), indicating that the chain mediating role of this path is positively significant. That is, the acquisition of information on food safety online can enhance risk perception by improving the public’s information credibility, thus affecting food risk prevention behaviour. Therefore, H5 is supported.

**Table 4:** Mediating effect test between information credibility and risk perception

Mediation path	Effect size	SE	95%CI	
			Lower limit	Upper limit
**X**→**M**_**1**_→**Y**	0.007	0.003	0.002	0.014
**X**→**M**_**2**_→**Y**	0.043	0.007	0.029	0.057
**X**→**M**_**1**_→**M**_**2**_→**Y**	0.002	0.001	0.001	0.003

Note: X indicates online food safety information acquisition, Y indicates food risk prevention behavior, M_1_ indicates information credibility, and M_2_ indicates risk perception

## Discussion

### Online food safety information acquisition is an important factor affecting food risk prevention behaviour

This study used propensity value matching to address potential selectivity bias and found that obtaining online food safety information significantly increases individuals’ food risk preventive behaviour. That is, the more frequently online food safety information is obtained, the more likely individuals are to adopt food risk preventive behaviour. When facing issues that have not yet been personally experienced, individuals are particularly susceptible to the influence of media information [70]. In crisis and risk situations, mass communication cultivates the public’s cognition and behavioural tendencies [71]. Social media can also produce this cultivation effect [72], making cognition, attitude, behaviour and other aspects change [73]. The internet, as a major source of information, can raise public awareness of food safety and improve food safety [74–76]. However, it is necessary to be vigilant about the “social amplification of risks” effect of the internet. Some online media not only present uncertain food risk information but also provide inaccurate, incorrect, or misleading information to catch the eye [77]. This can easily lead to public panic and produce a “vicarious traumatization” effect on non-first-hand experience, resulting in an irrational herd phenomenon. For government agencies, these challenges may be particularly severe [78]. Given the strong impact of online food safety information acquisition, online media should enhance their agenda-setting capabilities and expand the coverage and dissemination of correct food safety information on internet platforms. First, efforts should be made to plan topics that highlight the “acquisitionibility” framework, emphasizing the individual value of adopting food risk preventive behaviours. Second, the news media should establish an “action” framework, unite with opinion leaders in professional fields, popularize science effectively, comprehensively and credibly, and provide food safety knowledge related to the daily life of the public to improve the public’s awareness and ability to avoid food risks.

### The impact of topic attention difference reflects the group difference in the impact of online food safety information acquisition

This study found that internet users with high degrees of topic attention can focus their limited personal attention on the acquisition and reading of food safety information, which significantly increases relevant preventive behaviour. Users with low topic attention do not spend time on food safety information, and their input and effort motivation are low. They quickly browse or skip food safety information. Hence, relevant preventive behaviours are not affected by the acquisition of information on the internet.

These research findings further enrich relevant research at the theoretical level. Previous research has focused on the prediction of the degree of public attention to portal news and food information in social media on the public’s risk perception [79], with few heterogeneous effects of the degree of attention to research topics. At the practical level, the online media and the government should be reminded that the type and content of food safety news reports should differ from person to person to ensure that everyone receives accurate and timely information, which allows people to take preventive actions to protect their health and safety.

For the general public, their attention to the issue is usually affected by the degree of information intervention [80]. Zaichkowsky (1994) [81] proposed that the degree of individual information intervention can be measured with regard to the important, relevant, meaningful, and worthwhile dimensions. When food safety events occur, the news reports on online media and the response of the government can be formulated to emphasize the high relevance of food safety and the importance of taking preventive measures. These actions will improve the public’s attention to food safety information. In practice, we can also use big data technology to identify groups with low attention to food safety information among internet users and use algorithm recommendation technology to strengthen their food risk experience to encourage them to take corresponding preventive measures to protect their health and safety.

### Information credibility and risk perception are important psychological motivations for online food safety information acquisition to affect food risk prevention behaviour

This study found that information credibility plays a positive mediating role between online food safety information acquisition and food risk prevention behaviour. This is consistent with the research conclusions of Martins et al. (2018) [82]: information with high reliability is more easily accepted by users and can change their behaviour. The elaboration likelihood model (ELM) suggests that whether the audience must undergo careful and fine processing for attitude change can be divided into a central path and a marginal path [83]. The attitude formed by the central path is more stable than that of the marginal path. In the internet environment, the ELM model suggests that the quality of information content is the central path and the credibility of information sources is the marginal path [84]. In this regard, online media can report food risk events by adhering to the strategy of primarily improving the quality of information content and supplementing it with improvements to the credibility of information sources.

With regard to the quality of information content, the Internet User Information Adoption Model suggests that the quality of information content can be judged from four measurement criteria: accuracy, integrity, timeliness, and relevance [85]. To this end, online media should report the content of events related to the public’s immediate interests in a timely, scientific, comprehensive and complete manner with high information quality for the release of information on food safety risk events. Media should reject the “eyeball effect”, sensationalism and quoting out of context, thereby eliminating the public’s sense of uncertainty and helping the public form stable food risk prevention behaviours.

In terms of information source credibility, this study found that with regard to the credibility of information sources, institutional microbloggers are more reliable than individual microbloggers, and professional opinion leaders are more reliable than social celebrities [86]. When a food safety event occurs, online media and the government should invite research institutions and experts to interpret the event. These actions will increase the credibility of the information source and reduce the instability of the edge path and sleeper effect.

Moreover, this study found that risk perception plays a positive mediating role between online food safety information acquisition and food risk preventive behaviour. In other words, online food safety information acquisition can indirectly affect the public’s relevant preventive behaviour by influencing their risk perception. This research result is consistent with the existing results related to crisis and risk communication [87]. However, it should be noted that compared with indirect effects, online food safety information acquisition has stronger direct explanatory power for preventive behaviours. This further supports Perse’s view that in crisis situations, the influence of the direct effect model based on traditional magic bullet theory is enhanced [88], and users show stronger information compliance and action response to food safety information.

### From single to continuous: the serial mediating roles of information credibility and risk perception

Significantly, this study also found that information credibility and risk perception play a serial mediating role between online food safety information acquisition and food risk prevention behaviour. Previous studies mainly focused on the action mechanism of single mediating variables or multiple parallel mediating variables, and lack of exploration of the interaction mechanism of mediating factors. In practice, the interaction between different psychological factors will have different impacts on individual behaviours, and the implicit internal cognition is closely related to the explicit external behaviour. The chain mediation model can provide a more comprehensive and reasonable explanation for individual behavioural preferences. Therefore, this study innovatively proposes that the two should be seen as a continuous reaction process, because the stimulation effect of online food safety information acquisition is not only realized through one of the information credibility or risk perception, but more likely to affect the participating behaviour through the transmission and joint effect between the two. Information credibility positively affects risk perception, improves the public’s susceptibility and severity cognition of food risks, and further promotes the public to adopt food risk prevention behaviour. This fully reveals the “black box” mechanism of online food safety information acquisition affecting food risk prevention behaviors, and builds a theoretical chain of “information acquisition - information credibility - risk perception - prevention behaviour”. The relevant theory is extended effectively and provides a reference paradigm for the subsequent research.

Although the above research conclusions help to establish a possible path from the acquisition of information on food safety online to the prevention of food risk, there are still some limitations. First, this study was a cross-sectional study. The research logic was to first deduce and infer the causal relationship and mechanism between online food safety information acquisition and food risk prevention behaviour from the level of the PADM and then use the survey data of the Taiwan Communication Survey Database to confirm or falsify the research hypotheses. Second, the secondary data used in this study limited our choice and setting of some variables, which may have had an impact on the final research results. Third, this study only explored the impact of online food safety information acquisition on preventive behaviour at the individual level but failed to include structural factors at the macro level.

## Conclusions

Food safety is a significant health issue that people face daily. An increase in the frequency of online food safety information acquisition will significantly increase individuals’ food risk prevention behaviour. However, only users with high concern about the issue will be affected. The food risk prevention behaviour of users with low concern about the issue will not be affected by online food safety information acquisition. Further analysis showed that risk perception and information credibility both play a mediating role in the impact of online food safety information acquisition on food risk prevention behaviour. Moreover, the transmission and united effects between information credibility and risk perception play a distal mediating role. Our study findings can provide empirical evidence for risk managers and decision-makers to reevaluate the role of the internet in food risk management. Further studies should improve the operation of variables and explore the causal relationship and impact mechanism between online food safety information acquisition and food risk prevention behaviour in a more comprehensive and accurate way. In addition, follow-up studies can use the control experiment method, which may be more suitable for the test of causality.

## Data Availability

The datasets generated and/or analysed during the current study are available inthe [Taiwan Communication Survey] repository, [https://crctaiwan.dcat.nycu.edu.tw/index.asp].

## Footnotes

1 Please see the link for details: (1) Ministry of Health and Welfare: https://www.mohw.gov.tw/mp-1.html; (2) Food and Drug Administration: https://www.fda.gov.tw/TC/index.aspx; (3) Office of Food Safety of Executive Yuan: https://www.ey.gov.tw/ofs/.
